# Network-Based Differences in the Vaginal and Bladder Microbial Communities Between Women With and Without Urgency Urinary Incontinence

**DOI:** 10.3389/fcimb.2022.759156

**Published:** 2022-03-24

**Authors:** Rahel Nardos, Eric T. Leung, Erin M. Dahl, Sean Davin, Mark Asquith, W. Thomas Gregory, Lisa Karstens

**Affiliations:** ^1^ Division of Urogynecology, Oregon Health and Science University, Portland, OR, United States; ^2^ Division of Female Pelvic Medicine and Reconstructive Surgery, University of Minnesota, Minneapolis, MN, United States; ^3^ Division of Bioinformatics and Computational Biomedicine, Oregon Health and Science University, Portland, OR, United States; ^4^ Division of Arthritis and Rheumatology, Oregon Health and Science University, Portland, OR, United States

**Keywords:** urobiome, urinary microbiome, vaginal microbiome, urgency urinary incontinence, network analysis

## Abstract

**Background:**

Little is known about the relationship of proximal urogenital microbiomes in the bladder and the vagina and how this contributes to bladder health. In this study, we use a microbial ecology and network framework to understand the dynamics of interactions/co-occurrences of bacteria in the bladder and vagina in women with and without urgency urinary incontinence (UUI).

**Methods:**

We collected vaginal swabs and catheterized urine specimens from 20 women with UUI (cases) and 30 women without UUI (controls). We sequenced the V4 region of the bacterial 16S rRNA gene and evaluated using alpha and beta diversity metrics. We used microbial network analysis to detect interactions in the microbiome and the betweenness centrality measure to identify central bacteria in the microbial network. Bacteria exhibiting maximum betweenness centrality are considered central to the microbe-wide networks and likely maintain the overall microbial network structure.

**Results:**

There were no significant differences in the vaginal or bladder microbiomes between cases and controls using alpha and beta diversity. Silhouette metric analysis identified two distinct microbiome clusters in both the bladder and vagina. One cluster was dominated by *Lactobacillus* genus while the other was more diverse. Network-based analyses demonstrated that vaginal and bladder microbial networks were different between cases and controls. In the vagina, there were similar numbers of genera and subgroup clusters in each network for cases and controls. However, cases tend to have more unique bacterial co-occurrences. While *Bacteroides* and *Lactobacillus* were the central bacteria with the highest betweenness centrality in controls, *Aerococcus* had the highest centrality in cases and correlated with bacteria commonly associated with bacterial vaginosis. In the bladder, cases have less than half as many network clusters compared to controls. *Lactobacillus* was the central bacteria in both groups but associated with several known uropathogens in cases. The number of shared bacterial genera between the bladder and the vagina differed between cases and controls, with cases having larger overlap (43%) compared to controls (29%).

**Conclusion:**

Our study shows overlaps in microbial communities of bladder and vagina, with higher overlap in cases. We also identified differences in the bacteria that are central to the overall community structure.

## Introduction

Urgency urinary incontinence (UUI), which is defined as involuntary urinary leakage accompanied by or immediately preceded by urgency (Haylen et al., 2010), affects up to 30% of women with increasing risk with age (Coyne et al., 2012). Women are twice as likely to be affected by UUI as men. Many etiologies have been proposed for UUI ranging from neurogenic to idiopathic (less understood) causes. The underlying pathophysiologic factors that contribute to non-neurogenic UUI are thought to range from abnormal sensory function at the level of the urothelium or urethra (de Groat, 1997; Yamaguchi et al., 2007) to involuntary myogenic (detrusor) contractions (Abrams et al., 2002), altered brain function (Griffiths et al., 2009; Nardos et al., 2014; Nardos et al., 2016), and more recently, a shift in urinary bladder microbiome (Brubaker et al., 2014; Pearce et al., 2014; Pearce et al., 2015; Karstens et al., 2016). Other factors such as metabolic disorders (Bunn et al., 2015), affective disorders (Vrijens et al., 2015) and hormonal changes (Cody et al., 2012) have also been shown to be associated with this condition. These pathophysiologic factors are likely not mutually exclusive.

Recent advances in both culture and culture-independent techniques have made it possible to evaluate the role of microorganisms more thoroughly in health and disease. With these breakthroughs came the discovery that microbes contribute to human health more extensively than initially thought (Haiser et al., 2013; Fulde and Hornef, 2014; Neuman et al., 2015). Recent studies demonstrate that resident bacteria in the bladder may have a role in healthy bladder function (Siddiqui et al., 2011; Fouts et al., 2012; Wolfe et al., 2012; Khasriya et al., 2013; Lewis et al., 2013; Hilt et al., 2014; Wolfe and Brubaker, 2015). An alteration in the resident bladder bacterial community, on the other hand, may be associated with bladder disorders such as overactive bladder (OAB) with or without UUI (Brubaker et al., 2014; Pearce et al., 2014; Pearce et al., 2015; Karstens et al., 2016; Drake et al., 2017; Aragón et al., 2018) and interstitial cystitis (Siddiqui et al., 2012).

It is generally understood that the gut is the main source of urinary uropathogens by way of an intermediary, i.e., vagina. The female urogenital tract is particularly amenable to this migration theory given the anatomical characteristics and proximity of these organs to each other. The vaginal microbiome is therefore thought to play a critical role in contributing to bladder health in addition to its well-established role in maintaining a healthy vaginal environment (Ravel et al., 2011; Greenbaum et al., 2019). However, little is known about how these proximal ecosystems, i.e., microbiomes in bladder and vagina, relate to each other to affect bladder health or disease. In this study, we use a microbial network framework to understand the shift in the dynamics of interactions or co-occurrences of bacteria in the vagina and urinary bladder in women with and without UUI. Such application of network analysis methods allow us to understand the larger microbial community structures of the bladder and vagina and how they differ in the urogenital tract of women with and without UUI.

## Materials and Methods

This observational case–control study was conducted at the Oregon Health & Science University (OHSU) between 2016 and 2019. Study approval was obtained from the OHSU’s Institutional Review Board (IRB 00010729) as part of a larger effort to understand the role of urinary bladder microbiome in overactive bladder syndrome in women. Women between the ages of 45 and 85 were recruited both from the general population in the Portland area and through the Pacific Northwest Pelvic Floor Research Group, urogynecology clinical providers and researchers from the OHSU, Kaiser Permanente NW, and affiliated Portland-area hospitals. Participants were prescreened over the phone and those who met inclusion criteria completed their study visits at the OHSU’s Women’s Health Research Unit (WHRU).

We recruited twenty women with UUI (cases) and thirty women with normal bladder function (controls). The UUI group included women with daily urge-predominant incontinence confirmed on a three-day voiding diary, with urge-predominant leakage as determined by a Patient Perception of Intensity Urgency Scale (PPIUS) (Cartwright et al., 2010) score ≥3 (severe urgency that I could not postpone voiding) for >50% of total incontinence episodes on diary. The control group included female participants without a history of any UUI symptoms or frequent (>once a week) stress incontinence symptoms based on screening questionnaire and confirmed on three-day voiding diary. Participants were excluded if they had urinary retention with a baseline need for intermittent self-catheterization, known neurological diseases that could affect bladder function (stroke, multiple sclerosis, brain or spinal cord injury, myasthenia gravis), current pregnancy or lactation, history of pelvic radiation, current pelvic or bladder malignancy, symptomatic urinary tract infection detected on screening urinalysis and confirmed with culture (growth of >10^5^ colonies per ml), symptomatic pelvic organ prolapse (sensation of vaginal bulge), prior or current diagnosis of painful bladder syndrome, or a history of antibiotics in the previous two months.

All participants provided written consent and completed a demographic and health questionnaire and a three-day bladder diary. Participants were asked to score their urinary urgency on the bladder diary using the PPIUS. Participants also completed the International Consultation on Incontinence Questionnaire (ICIQ) (Avery et al., 2004), the Pelvic Floor Distress Inventory Urogenital Distress Inventory (UDI) (Barber et al., 2005) and the Overactive Bladder Questionnaire (OAB-q) (Coyne et al., 2002). These validated questionnaires assess urinary incontinence symptoms, impact of pelvic floor disorders on daily function, quality of life, symptom bother, and health-related quality of life, respectively. Finally, participants completed the Patient Global Impression of Severity (PGI-S) (Yalcin and Bump, 2003), the Patient Perception of Bladder Condition (PPBC) (Coyne et al., 2006) and the Beck’s Anxiety Inventory (Fydrich et al., 1992). During their study visit, urine was collected from the bladder using an aseptic technique with a urethral catheter by a trained and licensed practitioner. The total volume of urine was emptied, and urine specimens were aliquoted into sterile 50 ml conical tubes and stored at −20°C until further processing. Mid-vaginal swabs were also collected by a trained and licensed practitioner from the same participants on the same study visit. This was done by inserting a sterile cotton-tipped swab into the vagina, rotating the swab 360° five times, and letting the swab sit in mid vagina for 20 s. All specimens were handled in a sterile biosafety cabinet after collection.

### Statistical Analyses

Differences in clinical and demographic characteristics between UUI and controls were assessed with Student’s t-tests for normal and continuous characteristics, Kruskal–Wallis’ rank sum test for non-normal and continuous characteristics, and Fisher’s exact test for categorical data. The Shapiro–Wilk test was used to test for normality prior to testing. Clinical covariates that were found to be statistically different between UUI cases and controls were considered as covariates in downstream analyses. Data management, descriptive statistics, visualizations, and analyses were performed in R (version 3.6.1) (Team, 2017).

### Molecular Methods

Microbial DNA from vaginal swabs was extracted by vortexing swab heads in PowerBead tubes before centrifugation at 10,000*g* for 30 s at room temperature following the DNeasy PowerSoil DNA isolation kit (QIAGEN, Germany). Microbial DNA from the urinary bladder was extracted from microbial pellets formed from the centrifugation of 20–45 ml of urine at 10,000*g* for 30 min twice. DNA extraction was performed using the cultured cells protocol supplied with the DNeasy Blood and Tissue Kit (QIAGEN, Germany). The extracted DNA was quantified and quality checked at A260/A280 nm (Nanodrop, Thermo Fisher Scientific, USA) prior to amplification by polymerase chain reaction (PCR). No template controls and a mock microbial dilution series were also extracted with each protocol and subjected to amplification and sequencing.

Bacterial DNA was amplified by PCR using Golay barcoded primers which target the V4 region of 16S rRNA genes (Caporaso et al., 2012). Template DNA was amplified in triplicate using the GoTaq Hot Start Polymerase kit (Promega, USA). One microliter of template DNA and 1 μl of a unique barcoded reverse primer were added to 48 μl of master mix containing 1× colorless reaction buffer, 1.5 mM MgCl2, 0.2 mM dNTPs, 0.2 mM forward primer, and 1.25 U of polymerase enzyme. The reaction volumes were placed in a thermocycler and run through the following conditions: 94°C for 3 min (initial denaturation), followed by 35 cycles of 94°C for 45 s (denaturation); 55°C, 40 s (annealing); 72°C, 1.5 min (extension); with a final extension at 72°C for 10 min.

Ten microliters of each product were used to verify amplification by gel electrophoresis on a 2% agarose gel. Replicates yielding visible bands at 382 bp were pooled together and purified following the QIAquick PCR Purification kits (QIAGEN, Germany) provided protocol. Purified products were again quantified, and quality checked at A260/A280 nm (Nanodrop, Thermo Fisher Scientific, USA). Products were diluted to 10 ng/μl, and 5 μl of each sample were pooled together for sequencing on the Illumina MiSeq sequencer (Illumina, USA).

### Sequence Processing

Illumina sequence reads were processed using DADA2 (version 1.4.0) (Callahan et al., 2016) to yield amplicon sequence variants (ASVs), using default parameters unless otherwise noted. Briefly, reads were trimmed 10 bases from the 5’ end for both forward and reverse reads, and the 3’ ends were truncated to 240 and 160 bases, respectively. Chimeric sequences were identified and removed by taking a consensus across samples using the removeBimeraDenovo function. Taxonomy was assigned using the RDP classifier (Wang et al., 2007) with the Silva database (version 132) as implemented in the assignTaxonomy function. For further manipulations, we used Phyloseq (version 1.28.0) (McMurdie and Holmes, 2013) and several other R packages. ASVs were agglomerated to the genus taxonomic rank for downstream analyses. For diversity analyses, vaginal and bladder microbiome sequence variants were rarefied without replacement to 15,000 reads per sample and 2,500 reads per sample, respectively. Performed separately for vaginal and urine samples, genera that contributed greater than 5% of the total of at least one sample were considered for further analysis. Identification and removal of contaminant sequences was performed on urinary bladder microbiome samples using the Decontam (version 1.4.0) (Davis et al., 2018), using the frequency classification with a threshold of 0.3. Decontam was also performed on vaginal samples using the threshold of 0.5. Phylogenetic trees were constructed by generating a neighbor-joining tree based on a multiple sequence alignment as implemented with default parameters in DECIPHER (version 2.14.0) (Wright, 2016) and Phangorn (version 2.5.5) (Schliep, 2011).

### Microbiome Analyses

Stacked bar plots based on sequence abundance were produced for the vaginal and bladder microbiome samples using Microshades (Dahl et al., 2021). Weighted UniFrac distance was calculated between samples, and the updated Ward’s minimum variance method was used for agglomerative hierarchical clustering (Murtagh and Legendre, 2014) with complete linkage using hclust “ward.D2”. The clustering dendrogram was cut based on the silhouette clustering metric, a measure assessing the similarity of within-cluster points with other cluster points. The silhouette clustering metric was calculated for potential clusters of 2 through 6, and the largest value was used as the optimal number of clusters. A dendrogram was used to visualize the hierarchical clustering relationships *via* the Dendextend (version 1.13.4) (Galili, 2015).

Alpha and beta diversity were calculated for the UUI case and control samples using Phyloseq (McMurdie and Holmes, 2013), Vegan (version 2.5.6) (Oksanen et al., 2019), and Microbiome (version 1.6.0) (Lahti and Shetty, 2017) R packages. Alpha diversity was assessed with the observed number of taxa, Pielou’s evenness index (Pielou et al., 2007), and inverse Simpson index. Beta diversity between subject samples was calculated using the Bray-Curtis, weighted UniFrac and unweighted UniFrac (Lozupone and Knight, 2005) distance measures using the distance function in the Phyloseq, visualized using principal coordinates analysis (PCoA), and assessed with PERMANOVA (Anderson, 2014) for significance using the adonis function in the Vegan package.

### Network Analyses

SparCC was used to construct microbial networks. SparCC accounts for the compositional nature of 16S rRNA data by performing a linear Pearson correlation on log-ratio transformed data (Friedman et al., 2004). This transformation is beneficial because it retains the true abundance values as a ratio, which are independent of other taxa included in the data, and the transformation can take any value rather than being constrained to a fixed abundance. The SparCC method was performed as implemented in the sparcc function with default parameters in the SpiecEasi package (version 1.0.7) (Kurtz et al., 2015). Network analyses were performed using the R packages tidygraph (version 1.2.0) (Pedersen, 2020), with the underlying functionality of igraph (version 1.2.5) (Csardi and Nepusz, 2006), and visualized using the R package ggraph (version 2.0.3) (Pedersen, 2021). Community detection was performed using the InfoMap community detection algorithm, which minimizes the expected description length of a random walker along the network, as implemented in cluster infomap in the R package igraph (Rosvall and Bergstrom, 2008).

A permutation analysis was used on all UUI case and control vaginal microbiome data to determine a correlation threshold by shuffling the sample labels for each genus in a pairwise comparison prior to calculating SparCC correlations. A similar permutation was performed separately on the bladder microbiome data. This permutation analysis generates a null distribution of correlations from which to identify a threshold of correlations for downstream analyses. A permutation of 1,000 trials was performed and a threshold of the top 5% of the null distribution was used to determine a correlation cut off for each the vaginal microbiome (correlations >0.23) and bladder microbiome data (correlations >0.22). Only positive correlations were considered for the network analysis.

## Results

The study included twenty women with UUI and thirty women without UUI (controls). Women in both groups were similar in age, menopause status, estrogen use, number of vaginal deliveries, and race (p >0.05) (**Table 1**). There was also no significant difference in clinical history such as IBS, history of pelvic surgery, diabetes, or current tobacco use. However, women with UUI were more likely to have had a history of recurrent UTIs compared to controls (p = 0.007). Women with UUI were also significantly more likely to score higher on Beck’s Anxiety Inventory (10.6 ± 12.0) compared to controls (2.6 ± 2.6) (p <0.001) even though they did not report a higher incidence of anxiety diagnosis in their medical history.

**Table 1:** Participant demographics and comorbidities.

	UUI (N = 20)	Control (N = 30)	p-value
Age (years)	64.2 ± 10.5	57.9 ± 10.4	0.04
Body mass index (kg/m^2^)	29.25 [25.93, 32.8]	25.4 [23.2, 28.3]	0.005
Menopause status			0.32
Premenopausal	3 (15%)	9 (30%)	
Postmenopausal	17 (85%)	21 (70%)	
Any Estrogen use	9 (45%)	7 (23%)	0.22
Race			0.68
White	18 (90%)	27 (90%)	
Non White	2 (10%)	3 (10%)	
Vaginal delivery (Yes)	11 (55%)	17 (47%)	1.00
Number of vaginal deliveries	2.00 [1.50, 3.00]	2.00 [1.00, 3.00]	0.24
History of diabetes	4 (20%)	6 (7%)	0.14
Smoking (current)	1 (5%)	0 (0%)	0.40
Has history of recurrent UTI	5 (25%)	0 (0%)	0.007
History of Anxiety	4 (20%)	4 (13%)	0.27
Beck’s Anxiety Inventory Score	7.50 (2.5, 15.25)	2.0 (1.00, 3.00)	0.007
History of IBS	4 (20%)	2 (10%)	0.28
History of Pelvic Floor Surgery	7 (35%)	11 (37%)	1.00

Student’s t-test was performed on continuous, normally distributed data and displayed with mean and standard deviation. The Kruskal–Wallis test was performed on continuous, non-normally distributed data and displayed with median and the interquartile range. The Fisher’s Exact test was performed on categorical data and counts reported as number of individuals with corresponding demographic or condition.

As expected, the UUI group had higher scores on clinical symptom and symptom bother questionnaires related to incontinence (UDI, OAB-q, ICIQ, p <0.001, **Table 2**). The higher UDI-6, OAB-q symptom bother, and ICIQ scores for the UUI cohort indicate the severity of symptom bother and disability in this particular population. Similarly, the lower OAB-q health-related quality of life scores for the UUI cohort indicates a lower quality of life in the UUI population. Objective measures of incontinence are captured by a three-day bladder diary which showed that 60% of the UUI population had daily urge leaks and 15% had daily stress leaks while the control group did not have any urge or stress leaks. There was no significant difference in nocturia or daytime urinary frequency between the two groups.

**Table 2:** Participant bladder symptoms.

	UUI	Control	p-value
Urogenital distress inventory (UDI-6 Short Form)	5.5 [4.8, 9.0]	0.0 [0.0, 0.0]	<0.001
Overactive Bladder Questionnaire (OAB-q) symptom bother	45.0 [40.0, 70.0]	6.7 [3.3, 15.0]	<0.001
OAB-q health-related quality of life	66.9 [45.8, 78.9]	98.5 [95.8, 100.0]	<0.001
International Consultation on Incontinence Questionnaire (ICIQ)	10.5 [8.0, 14.3]	3.0 [0.0, 3.0]	<0.001

This table summarizes bladder symptoms, assessed by validated pelvic floor questionnaires. Statistics performed by Kruskal–Wallis, comparing UUI cases to controls and displayed with the median and IQR. IQR, interquartile range.

The 16S rRNA amplicon sequencing resulted in a mean sequencing read depth of 45,005 reads per sample (range 15,201–69,787) for vaginal samples and 38,941 reads per sample (range 2,319–137,986) for urine samples. There were no significant differences in the number of reads per sample between the UUI and control groups (p = 0.7 vaginal samples, p = 0.2 urine samples). After processing and filtering as described in the methods, the vaginal sequencing resulted in classification of 8 phyla, 12 classes, 18 orders, 31 families, and 66 genera. Urine sequencing resulted in classification of 14 phyla, 21 classes, 43 orders, 70 families, and 131 genera.

### Vaginal Microbiome

**Figure 1:**
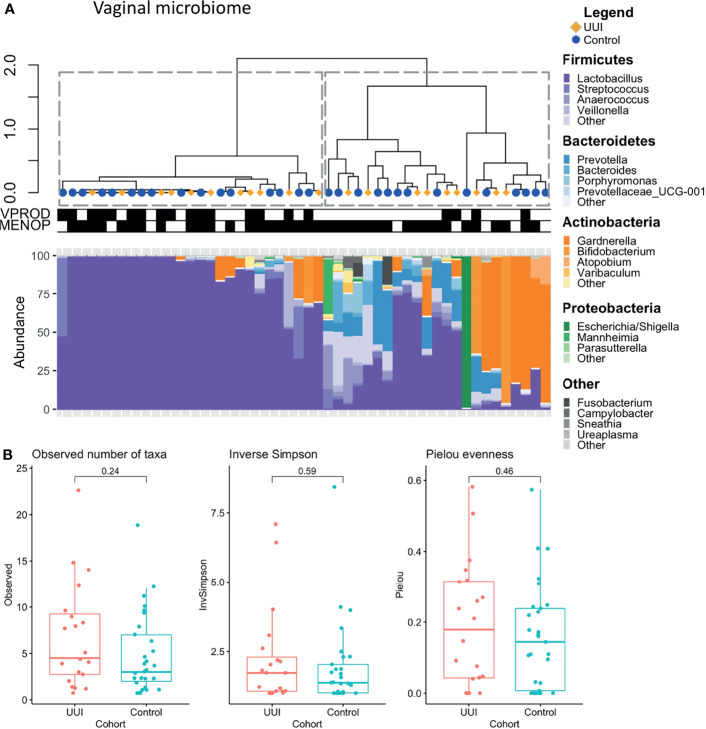
**(A)** Clustering of vaginal microbiome is associated with use of vaginal products. Hierarchical clustering (top) was performed using the Ward’s minimum variance method on weighted UniFrac distances between samples. Stacked bar plots (bottom) show relative abundance of vaginal microbiome of women with and without UUI. Dotted lines outline clusters that were chosen based on the Silhouette metric. Black bars underneath annotate for clinical features being TRUE. Vaginal product use was significantly associated with vaginal microbiome cluster (p = 0.004), menopausal status was not (p = 1.0, Fisher’s exact test). Cases are labeled with yellow diamonds and controls are labeled as blue squares. VPROD, any vaginal product use; MENOP, post-menopausal. **(B)** Women with and without UUI do not differ in vaginal microbiome diversity. Alpha diversity is visualized using box-and-whisker plots and measured using observed number of taxa (genera, p = 0.24), inverse Simpson Index (p = 0.59), and Pielou evenness index (p= 0.46). A generalized linear model was used to adjust for age, BMI, and menopause-estrogen status.

We identified two distinct clusters of vaginal microbiome profiles (silhouette score 0.63, **Figure 1A**). One cluster was dominated by the genus *Lactobacillus*, while the second cluster included microbiomes that were dominated by *Gardnerella*, *Bifidobacterium*, *Escherichia* or had no dominant bacteria and contained a mixture of *Anaerococcus*, *Prevotella*, *Escherichia*, *Gardnerella*, *Bifidobacterium*, or other genera. We tested the relationship between these vaginal microbiome clusters and demographic/clinical characteristics and found no association with cohort status (UUI vs. control) (p = 1.0) or menopausal status (p = 1.0). However, the clustering was associated with vaginal product use, with more participants in the *Lactobacillus*-dominated clusters reporting use of vaginal products (p = 0.004). Vaginal products were defined as any use of vaginal medication or suppository, douche, feminine spray, spermicide, or personal lubricant in the week prior to collection of vaginal microbiome samples. There was no difference in either alpha diversity (**Figure 1B**) or beta diversity in the vaginal microbiome of women with and without UUI (**Supplemental Figure 1** PERMANOVA p = 0.71, adjusted for age, BMI, menopause status, and estrogen use). Of the 16 participants who reported use of vaginal products, 13 reported vaginal estrogen use as the vaginal product. Therefore, we excluded vaginal product from the adjusted model and only included estrogen use.

**Figure 2:**
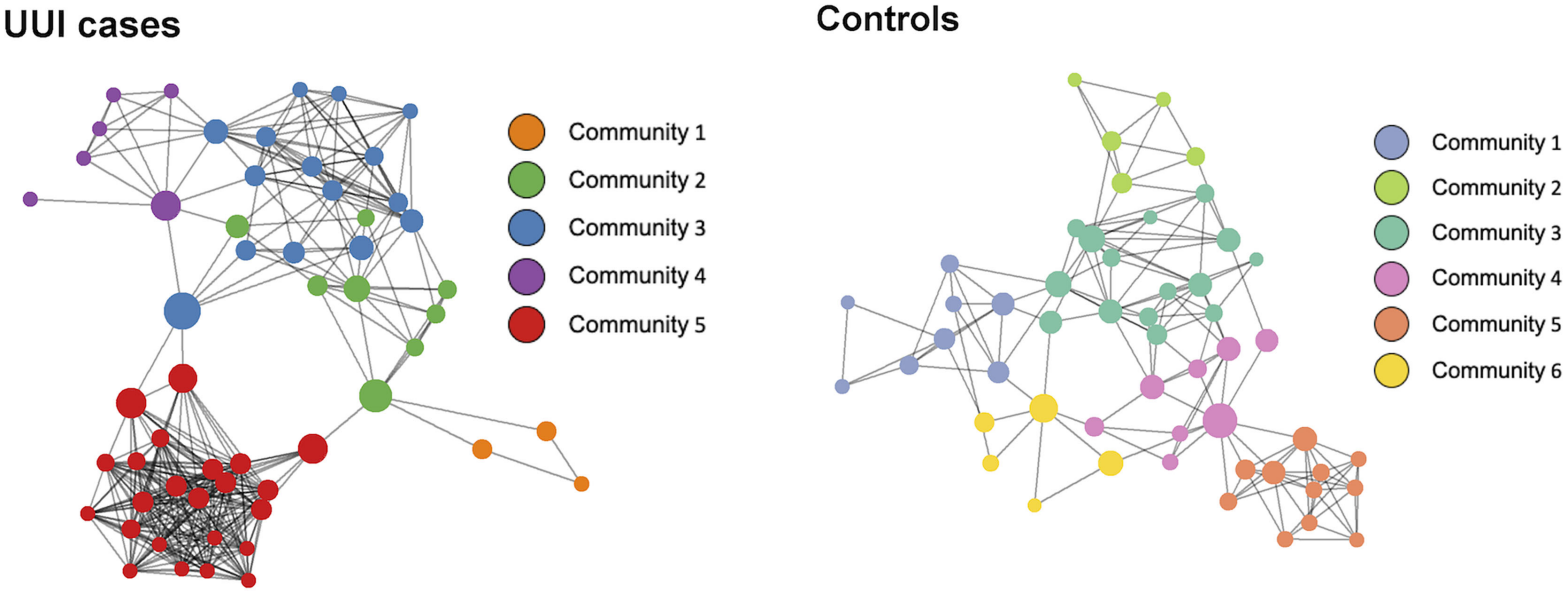
Network visualization of vaginal microbiomes. Each microbiome co-occurrence network, one for the UUI group (left) and one for the control group (right) consists of several bacterial genera (circles, colored by communities of bacteria identified using the InfoMap algorithm and sized by betweenness centrality value) that are connected to other bacterial genera by co-occurrence (edges/lines connecting circles, identified by SparCC correlation).

For each group, we inferred a microbiome-wide interaction network based on the bacterial genera in the vaginal microbiome (**Figure 2** and **Table 3**). Of note, interaction in this correlation-based network does not indicate physical or biochemical interactions among microbes. There was a similar number of genera in the microbiome networks of both UUI and control groups (55 genera versus 52 genera respectively) but more unique bacterial co-occurrences (pairs of bacteria that co-occur) in the UUI group compared to controls (343 associations versus 152 associations respectively). The number of clusters in the vaginal network were similar between UUI and controls, where the UUI network clustered into 5 subgroups of bacteria and controls clustered into 6 subgroups.

**Table 3:** Summary of metrics for vaginal microbiome networks.

	Interpretation	UUI	Control
Number of nodes	Space of co-occurring bacteria to consider	55	52
Number of edges	Number of co-occurrence relationships	343	152
Modularity	Measure of community detection	0.45	0.57
Normalized connectance	Complexity of system	0.23	0.11

We further explored the presence of a central bacteria in each of the vaginal networks using the betweenness centrality measure (**Table 4**). Central bacteria have high betweenness centrality, i.e., in the path between most other bacterial networks, potentially leveraging higher influence over network stability. *Bacteroides* were the central bacteria in the vaginal microbiome-wide network of control samples with the highest centrality measure of 0.39, followed by *Lactobacillus* (betweenness centrality of 0.19). In the UUI group, *Aeroccocus* were the central genus in the vaginal microbiome-wide network with a centrality measure of 0.33, followed by *Streptococcus* (betweenness centrality of 0.22). *Lactobacillus* were not found to be in the top six most central bacteria in the vaginal microbiome-wide network of UUI subjects. We further explored the surrounding bacterial associations to the central bacteria in the UUI group (**Table 5**). These top associated bacteria include *Actinomyces*, *Staphylococcus*, *Helcococcus*, *Streptobacillus*, *Prevotellaceae*, *Gardnerella*, and *Bacteroides.*

**Table 4:** Betweenness centrality measured of key bacteria in the vaginal microbiome network (defined by bacteria with a betweenness score >0.10).

Genus	UUI	Control
*Bacteroides*	0.14	0.39
*Aerococcus*	0.33	0.07
*Streptococcus*	0.22	0.07
*Lactobacillus*	0.00	0.19
*Mannheimia*	0.16	0.04
*Prevotellaceae_UCG-001*	0.16	0.01
*Gardnerella*	0.15	0.06
Anaerococcus	0.02	0.15
*Fusobacterium*	0.00	0.14
*Staphylococcus*	0.06	0.12

**Table 5:** Top bacteria correlated with *Aerococcus* in the UUI vaginal network.

Genus	Correlation
*Actinomyces*	0.46
*Staphylococcus*	0.41
*Helcococcus*	0.39
*Streptobacillus*	0.39
*Prevotellaceae*	0.36
*Gardnerella*	0.36
*Bacteroides*	0.24

### Bladder Microbiome

**Figure 3:**
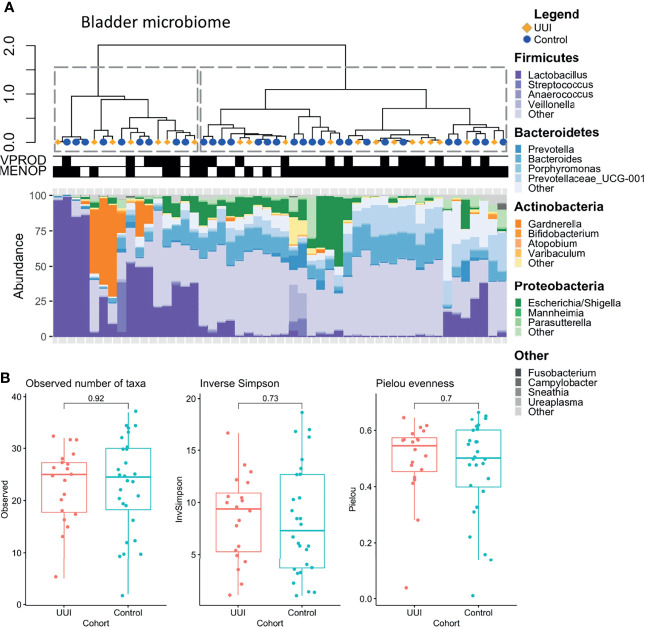
**(A)** Clustering of bladder microbiome is associated with menopausal status. Hierarchical clustering was performed using the Ward’s minimum variance method on weighted UniFrac distances between samples. Dotted lines outline clusters that were chosen based on the Silhouette metric. Black bars underneath annotate for clinical features being true. Menopausal status was associated with bladder microbiome cluster (p = 0.01), but vaginal product use was not (p =1.0, Fisher’s exact test). Cases are labeled with yellow diamonds and controls are labeled as blue squares. MENOP, post-menopausal. **(B)** Women with and without UUI do not differ in bladder microbiome diversity. Alpha diversity is visualized using box-and-whisker plots and measured using observed number of taxa (genera, p = 0.92), inverse Simpson Index (p = 0.73), and Pielou evenness index (p= 0.70). A generalized linear model was used to adjust for age, BMI, and menopause-estrogen status.

For the bladder microbiome, we identified two distinct clusters using the silhouette metric which is similar to vaginal microbiome (highest silhouette score of 0.50, **Figure 3A**). One cluster had a diverse mix of bacteria, namely, *Bacteroides*, *Escherichia*, *Blautia*, *Faecalibacterium*, *Lachnospiraceae*, *Prevotellaceae*, and others, while the other cluster was primarily dominated by *Lactobacillus* or *Gardnerella*. We tested for associations between the microbiome clusters and clinical/demographic characteristics and found no relationship between cohort status (UUI vs control, p = 0.76) or vaginal product use (p = 0.76). However, menopausal status did have a significant association with the clusters (p = 0.01), with the diverse microbiome cluster being more associated with samples from postmenopausal women. We did not find any significant differences in alpha diversity (**Figure 3B**) or beta diversity in the bladder microbiome of women with and without UUI (**Supplemental Figure 2**, PERMANOVA p = 0.23, adjusted for age, BMI, menopause status, and estrogen use).

**Figure 4:**
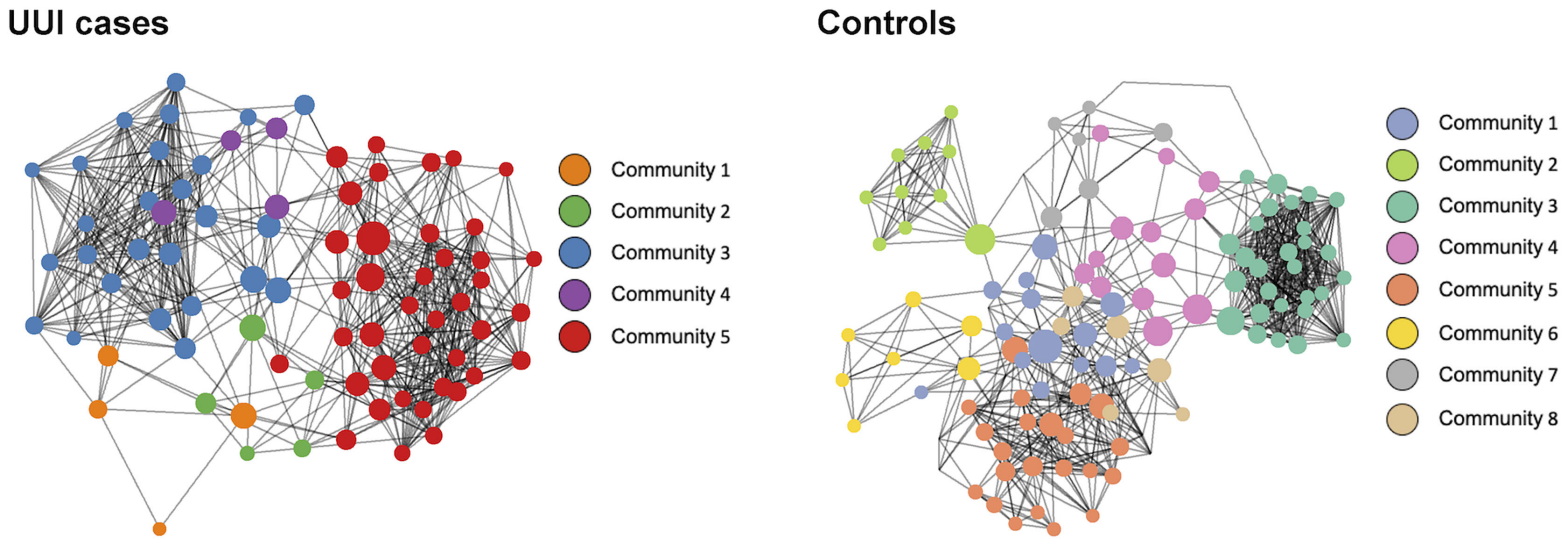
Network visualization of bladder microbiomes. Each microbiome co-occurrence network, one for the UUI group (left) and one for the control group (right) consists of several bacterial genera (circles, colored by communities of bacteria identified using the InfoMap algorithm and sized by betweenness centrality value) that are connected to other bacterial genera by co-occurrence (edges/lines connecting circles, identified by SparCC correlation).

Similar to the vaginal microbiome analysis, we inferred microbiome-wide interaction network for each cohort group (UUI and control) independently (**Figure 4** and **Table 6**). The basic structure of the bladder microbiome network showed five clustered subgroups of bacteria for women with UUI and eight clusters for controls. Visually and quantitatively using modularity and connectance, we see that the control bladder microbial network is more clustered into smaller microbial groups. The UUI network had fewer genera in the connected network (93 genera in UUI versus 135 genera in controls) and fewer unique bacterial co-occurrences (624 associations in UUI versus 763 associations in controls).

**Table 6:** Summary of network metrics for bladder microbiome networks.

	Interpretation	UUI	Control
Number of nodes	Space of co-occurring bacteria to consider	93	135
Number of edges	Number of co-occurrence relationships	624	763
Modularity	Measure of community detection	0.46	0.60
Normalized connectance	Complexity of system	0.15	0.09

We explored key urinary bacteria in the UUI and control bladder microbial networks using the betweenness centrality measure. Unlike the vaginal microbiome, we found that *Lactobacillus* genera were the central bacteria with the highest centrality measure in both UUI and controls (**Table 7**). Because *Lactobacillus* are central to both UUI and control networks, we explored the microbial associations between the *Lactobacillus* and other genera in each cohort. We found 22 unique genera in UUI and 20 unique genera in the controls that associated with *Lactobacillus* in their respective networks. Both networks shared a total of six common genera that associated with *Lactobacillus*–*Campylobacter, Dialister*, *Gardnerella*, *Prevotellaceae_NK3B31_group*, *Ureaplasma*, and *Varibaculum*. Among the unique associated genera in UUI, we found that at least a quarter of them give rise to species known to be uropathogens associated with urinary tract infections. These include *Aerococcus*, *Corynebacterium*, and *Escherichia/Shigella* (**Table 8**).

**Table 7:** Betweenness centrality measures of key bacteria in the bladder microbiome network (defined by bacteria with a betweenness score >0.10).

Genus	UUI	Control
*Lactobacillus*	0.14	0.20
*Corynebacterium*	0.01	0.15
*Bifidobacterium*	0.02	0.13
*Akkermansia*	0.00	0.13

**Table 8:** Top bacteria correlated with *Lactobacillus* in the urinary network.

Genera associated in both groups	
Genus	Correlation
*Campylobacter*	0.43
*Dialister*	0.37
*Gardnerella*	0.28
*Prevotellaceae_NK3B31_group*	0.34
*Ureaplasma*	0.34
*Varibaculum*	0.31
**Genera unique to UUI group**	
Genus	Correlation
*Actinotignum*	0.42
*Aerococcus*	0.32
*ASF356*	0.39
*Corynebacterium*	0.33
*Erysipelotrichaceae_UCG-003*	0.37
*Escherichia/Shigella*	0.31
*Flavonifractor*	0.33
*Intestinimonas*	0.30
*Jonquetella*	0.42
*Lachnospiraceae_NK4B4_group*	0.35
*Luteibacter*	0.32
*Marvinbryantia*	0.31
*Meiothermus*	0.40
*Oscillibacter*	0.41
*Paludibacter*	0.30
*Prevotella*	0.30
*Ruminococcaceae_NK4A214_group*	0.31
*Ruminococcaceae_UCG-009*	0.24
*Ruminococcaceae_UCG-013*	0.32
*Sneathia*	0.30
*Tumebacillus*	0.38
*Tyzzerella*	0.24

### Vaginal and Bladder Microbiomes: Is There an Overlap?

**Figure 5:**
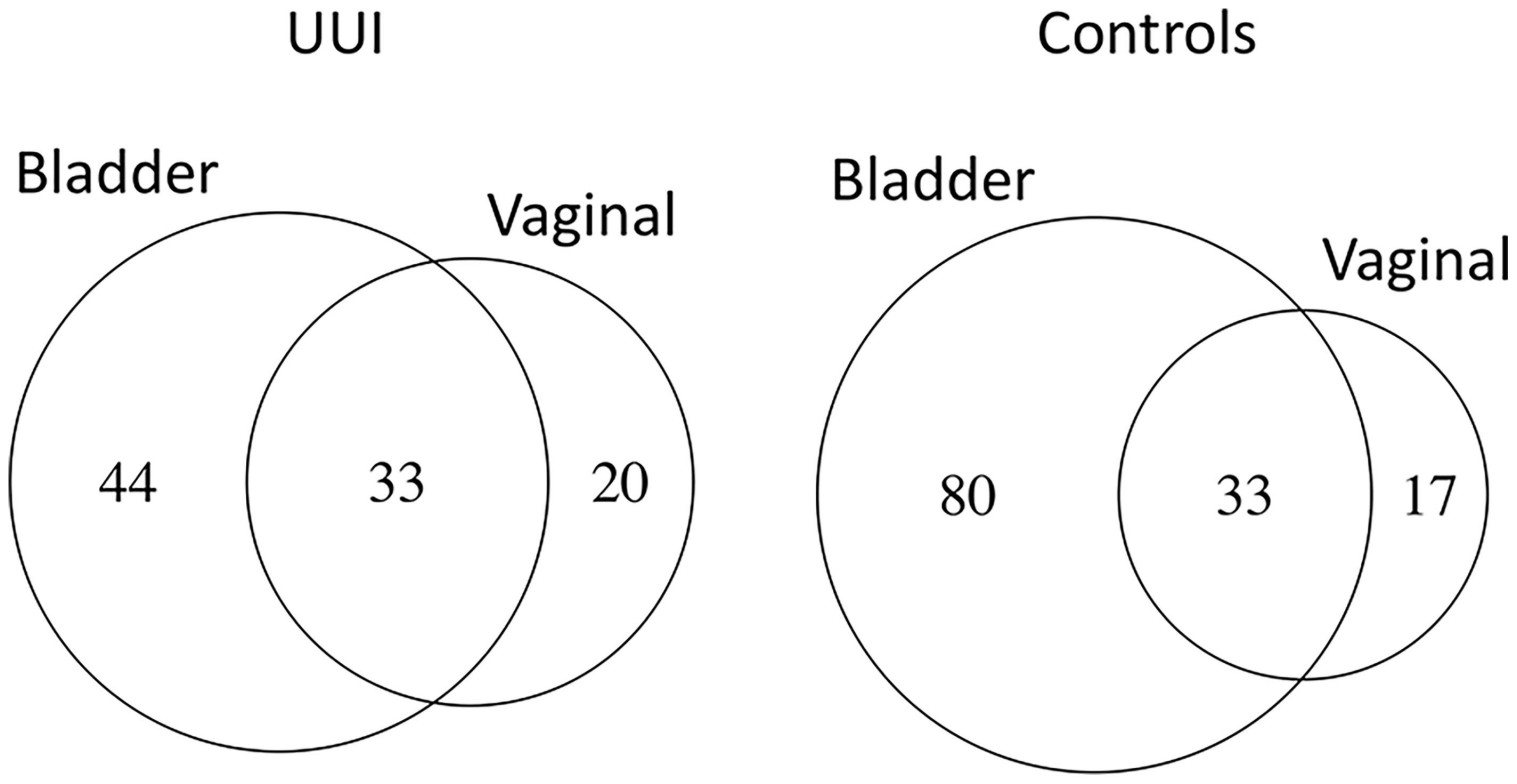
The number of shared bacterial genera between vaginal and bladder microbiomes of women with UUI differ from women without UUI. Women without UUI (controls) have more unique bacteria in the bladder microbiome.

Overall, more bacterial genera were identified from the bladder (131) compared to the vagina (66). The number of shared bacterial genera between the bladder and the vagina differed between women with and without UUI. Women with UUI have a larger number of shared bacterial genera (43%) between the two adjacent ecosystems compared to controls (29%) (**Figure 5** and **Table 9**). Among women with UUI, the top most abundant vaginal bacterial genera that were also present in the bladder include *Lactobacillus*, *Bifidobacterium*, *Gardnerella*, *Prevotella*, *Sneathia*, *Faecalibacterium*, *Varibaculum*, *Actinotignum*, and *Aerococcus.* Of these, only *Gardnerella* was more abundant in the bladder compared to the vagina. Other overlapping genera with higher median abundance in the bladder include *Bacteroides*, *Prevotellaceae_UCG-001*, and *Escherichia/Shigella.* Among controls, the most abundant vaginal bacterial genera that were also present in the bladder include *Lactobacillus*, *Gardnerella*, *Bifidobacterium*, *Atopobium*, *Prevotella*, *Bacteroides*, and *Streptococcus.* Of these, only *Bacteroides* was more abundant in the bladder compared to the vagina. Other top bladder genera with higher median abundance in the bladder compared to vagina include *Prevotellaceae_UCG-001*, *Actinomyces*, and *Faecalibacterium.* Irrespective of overall abundance in the vagina or bladder, the bacteria that has the highest overlap in median abundance in both ecosystems in the UUI group was *Gardrenella* (Vagina: 26.8, Bladder: 55.8) followed by *Lactobacillus* (Vagina: 69.6, Bladder: 6.3) where as in the control group, the bacteria with the highest overlap in median abundance were *Lactobacillus* (Vagina: 74.2, Bladder: 15.5) followed by *Gardrenella* (Vagina: 43.0, Bladder: 3.1).

**Table 9:** Bacteria shared across vaginal and bladder microbial communities in women with UUI and Controls, ranked by median abundance in the vaginal community.

UUI				Controls		
Bacteria	Vaginal median		Bladder median		Vaginal median	Bladder median
**Firmicutes**				**Firmicutes**		
*Lactobacillus*	69.56		6.27	*Lactobacillus*	74.24	15.46
*Faecalibacterium*	5.58		5.16	*Streptococcus*	4.04	1.49
*Aerococcus*	3.7		4.6	*Veillonella*	3.08	1.21
*Agathobacter*	2.49		3.56	*Peptostreptococcus*	2.18	0.88
*Lachnospira*	2.49		1.69	*Agathobacter*	1.5	2.91
*Ruminiclostridium*	2.22		6.62	*Faecalibacterium*	1.45	5.04
*Subdoligranulum*	2.2		3.06	*Roseburia*	1.28	1.84
*Ruminococcus*	2.14		2.48	*Enterococcus*	1.21	2.14
*Anaerostipes*	2.01		1.66	*Ruminococcus*	1.15	2.67
*Dialister*	1.77		1.88	*Lachnospiraceae NK4A136 group*	1.03	2.31
*Lachnospiraceae NK4A136 group*	1.31		2.57	*Ruminiclostridium*	1.02	3.48
*Fusicatenibacter*	1.23		3.18	*Blautia*	0.91	3.86
*Roseburia*	1.2		3.39	*Dialister*	0.85	1.33
*Streptococcus*	1.1		0.84	*Lachnospira*	0.85	1.81
*Phascolarctobacterium*	1.01		1.19	*Ruminococcaceae UCG-002*	0.81	1.56
*Blautia*	1		3.89	*Staphylococcus*	0.7	0.88
*Butyrivibrio*	0.9		1.04	*Anaerostipes*	0.61	1.58
*Lachnospiraceae ND3007 group*	0.83		0.97			
*Dorea*	0.75		1.16			
*Ruminococcaceae UCG-013*	0.66		1.26			
**Actinobacteria**				**Actinobacteria**		
*Bifidobacterium*	64.35		0.86	*Gardnerella*	43.04	3.09
*Gardnerella*	26.82		55.84	*Bifidobacterium*	31.7	0.99
*Varibaculum*	4.98		1.29	*Atopobium*	13.1	1.33
*Actinotignum*	4.11		0.8	*Actinomyces*	1.49	5.2
				*Corynebacterium*	0.89	0.71
				*Varibaculum*	0.86	0.57
**Bacteroidetes**				**Bacteroidetes**		
*Prevotella*	16.19		1.57	*Prevotella*	10.09	2.48
*Prevotellaceae Ga6A1 group*	2.21		2.83	*Bacteroides*	5.32	9.1
*Prevotellaceae UCG-001*	1.66		13.28	*Prevotellaceae NK3B31 group*	1.77	1.04
*Alistipes*	0.78		1.57	*Prevotellaceae UCG-001*	1.31	8.52
*Bacteroides*	0.71		16.39	*Alistipes*	0.81	2.24
**Proteobacteria**				**Proteobacteria**		
*Escherichia/Shigella*	1.8		4.63	*Escherichia/Shigella*	1.51	5.08
				*Salmonella*	1.11	2.32
**Tenericutes**				**Tenericutes**		
*Ureaplasma*	1.15		1	*Ureaplasma*	1.6	1.06
**Epsilonbacteraeota**				**Epsilonbacteraeota**		
*Campylobacter*	1.66		2.08	*Campylobacter*	1.4	1.87
**Fusobacteria**				**Verrucomicrobia**		
*Sneathia*	8.15		0.86	*Akkermansia*	2	2.9

## Discussion

It is generally accepted that the vagina acts as an intermediary between the gut and the bladder and may play a role in the pathogenesis of bladder conditions such as UTI (Czaja et al., 2009). It is also known that disruption of a normal microbiome environment in the vagina is associated with colonization by pathogenic organisms leading to disorders like bacterial vaginosis (Srinivasan et al., 2010), sexually transmitted infections (STIs) (van de Wijgert, 2017; Eastment and McClelland, 2018; Ziklo et al., 2018), and genital herpes infection (Shannon et al., 2017). It is however unclear if and how dysbiosis in the vagina plays a role in more chronic bladder conditions such as UUI.

In this study, we provide a detailed characterization of the differences in both bladder and vaginal microbiomes in a cohort of well-characterized women with and without UUI. More importantly, we go beyond traditional approaches to microbiome analysis by leveraging network-based analysis to look at underlying microbial community dynamics or interactions in these adjacent ecosystems. Network analysis provides a mathematical tool to understand complex systems such as identifying central bacteria that contribute to stability and resilience of ecosystems and their interactions with others in the network (Pielou et al., 2007).

In our study, women with UUI were similar to women without UUI in all clinical and demographic variables except for history of recurrent UTIs. Women with UUI were more likely to have a history of recurrent UTI which is not surprising given the known overlap in symptoms between UTI and UUI and the frequent misdiagnosis of UTI as a result (Nik-Ahd et al., 2018). There is also some evidence that overactive bladder may be associated with chronic low-grade colonization by bacteria that are commonly missed on routine cultures (Balachandran et al., 2016). These observations are the impetus for investigators, us included, to look at the role of bladder microbiomes in health and disease (Karstens et al., 2016; Brubaker and Wolfe, 2017; Drake et al., 2017; Aragón et al., 2018).

In our study, we did not see a significant difference in alpha or beta diversity in bladder or vaginal microbiomes of women with and without UUI. This is consistent with our own prior reporting (Karstens et al., 2016) but differed from others who reported increased diversity in UUI (Pearce et al., 2015; Thomas-White et al., 2016). Using silhouette analysis, we showed that the bladder and vaginal microbiomes formed two clusters of bacteria. In the vagina, one cluster was dominated by *Lactobacillus* and the other with mixed bacteria. Presence or absence of UUI did not impact clustering. The only clinical factor that was associated with this clustering was use of “any vaginal product” the week before collection of the vaginal specimen, with more vaginal product use noted in the *Lactobacillus*-dominated group. This observation is important to note and highlights the importance of environmental factors such as vaginal product use (douching, lubrication, estrogen cream etc.) that influence vaginal microbiome. This is consistent with the literature highlighting the dynamic nature of vaginal microbiomes and how these ecosystems can be perturbed by factors like menstruation, vaginal products, vaginal infections, hormonal status such as pregnancy, menopause, etc. (Srinivasan et al., 2010; Gajer et al., 2012; DiGiulio et al., 2015). Interestingly, the clustering in the vaginal microbiome was not associated with estrogen use *per se*. The small size of our study (with an even smaller number of women on estrogen therapy) does not allow us to make any definitive conclusion about the association of vaginal or systemic estrogen use and changes in vaginal or bladder microbiomes. This certainly needs further investigation given the promising evidence that vaginal estrogen improves OAB symptoms in post-menopausal women (Cardozo et al., 2004), reduces symptoms associated with genitourinary syndrome of menopause including the risk of UTI (Rahn et al., 2014), and that vaginal estrogen use may increase *Lactobacillus* in the urine of post-menopausal women (Thomas-White et al., 2020). This suggests that modulation of microbiomes is one likely mechanism of action for vaginal estrogens.

Similar to the vagina, silhouette analysis in the bladder microbiome identified two clusters. One cluster contains bladder microbiomes that have a higher proportion of *Lactobacillus* and *Gardnerella*, while the second cluster contains microbiomes with a mixed population of bacteria. This clustering in the bladder microbiome was associated with menopausal status in which post-menopausal women were more likely to have microbiomes without a dominant genus while premenopausal women tended to have microbiomes that were dominated by *Lactobacillus* or *Gardnerella*. This is consistent with prior report that shows higher *Lactobacillus* in the urine of premenopausal women compared to post-menopausal women (Curtiss et al., 2018). In our study, these dominant clusters in the bladder were not associated with presence or absence of UUI. This is in agreement with some prior work using similar-sized cohorts (Pearce et al., 2014), but different from other studies that found several clusters within UUI samples that were dominated by single bacteria such as *Lactobacillus* and *Gardnerella* (Pearce et al., 2015) and studies that identified bacterial community types that were different between women with and without mixed urinary incontinence (Komesu et al., 2018).

One of the unique aspects of this study was the use of network analysis to understand microbial interactions in the bladder and in the vagina. We identified two major microbiome interaction networks in both the vaginal and bladder samples. In the vaginal samples, we identified similar numbers of genera in each network for women with and without UUI. They also have similar numbers of clusters of subgroups in the microbiome-wide networks. However, women with UUI tend to have more unique bacterial associations or co-occurrences compared to controls. In the bladder on the other hand, cases have less than half as many network clusters compared to controls, suggesting that loss of smaller and more specialized microbiome networks may be a characteristic of UUI.

As part of our network analysis, we looked further to identify central bacteria that are defined by the highest betweenness score (centrality measure) and potentially play important roles in stabilizing the whole community. In network analysis, a node (which in our study represents a genus) that has maximum centrality can be thought of as a keystone taxon which maintains the network structure and potentially the function of the ecosystem. This is irrespective of whether or not it is the most abundant bacteria. In our study, we chose the threshold of 0.10 for our betweenness score because 90% of the values for both microbiome networks were lower than this threshold. In the vaginal microbiome network analysis, we found that *Bacteroides* and *Lactobacillus* were the two top central genera in control samples while *Aerococcus* was the central bacteria in women with UUI. *Bacteroides* as a central bacterium in women with normal bladder control is consistent with what we know about the role of *Bacteroides* in maintaining health in the urogenital tract. For example, delay in appearance or absence of *Bacteroides* has been observed in the guts of infants born by cesarean section compared to those delivered by the vaginal route, and this has been proposed as one of the etiologies for the higher predisposition of children born by cesarean section to disorders related to poor immune system maturation (Grönlund et al., 1999; Huurre et al., 2008). This seems to indicate that *Bacteroides* in the maternal birth canal (vagina) may have a key role for developing a healthy immune system that may persist in adults. *Lactobacillus*, the second most central vaginal bacteria in our control group, have been known to be a characteristic of a healthy vagina (Ravel et al., 2011; Drell et al., 2013). Our finding shows that beyond being the most abundant bacteria in the healthy vagina, *Lactobacillus* are also the central bacteria in the microbiome-wide network and thus may play a key role in the stability and function of this ecosystem. In our study, we did not perform a species-level analysis and thus are unable to explain the relevance of our findings on known vaginal community state types dominated by various species of *Lactobacillus*, such as *L. crispatus*, *L. gasseri*, *L. iners*, *bacterial vaginosis-associated bacteria* and *L. jensenii.*

In women with UUI, we found that the most central bacteria in the vaginal microbiome network is *Aerococcus*. Some species within this genus, such as *A. urinae*, are increasingly being recognized for their pathologic role in urinary tract disorders such as UTI, OAB, and UUI (Pearce et al., 2014; Kline and Lewis, 2016; Hilt et al., 2020). Among the top genera most associated with *Aerococcus* were *Actinomyces*, *Gardnerella*, *Prevotella*, and *Bacteroides. Actinomyces* species have been reported as associated with genitourinary actinomycosis (particularly in the setting of IUD use) and urinary tract actinomycosis (Huang and Al-Essawi, 2013; García-García et al., 2017). *Gardnerella* and *Prevotella* are well-known pathogens involved in bacterial vaginosis (Randis and Ratner, 2019). These findings seem to suggest that dysbiosis in the vagina characterized by changes in microbiome-wide community structure may be associated with urinary disorders like UUI.

In the bladder microbiome network analysis, *Lactobacillus* was the central genera in both UUI and control groups. Both groups shared *Lactobacillus* association with six common genera, but they each had several unique genera that did not overlap. Interestingly, many of the unique associations seen in women with UUI were with known uropathogens associated with UTI. It is important to note that in the bladder microbiome network analysis, what differentiates women with and without UUI is not the type of central bacteria (*Lactobacillus* for both*)* but rather its association with other bacteria in the network. This emphasizes the role of community network structures in health and disease and the need to go beyond quantification of relative abundance or diversity measures of microbiomes to understand dynamic ecosystems.

Looking at the similarities across the bladder and vaginal microbiomes, our study shows that women with UUI have a larger number of shared bacteria (43%) compared to women without UUI (29%). Interestingly, we found that although the two top bacteria that were most abundantly shared between these two ecosystems were the same between UUI and control samples (*Lactobacillus* and *Gardnerella), Gardnerella* was the most shared in the UUI subjects (24% overlap vs. 8.3%) while *Lactobacillus* dominated in the controls (17.3% overlap vs. 6.7%). Komesu et al. showed an overlap of 60% of the bacterial taxonomic units (genera) between the vagina and the bladder with the most abundant being the genus *Lactobacillus* (2020). Their study used younger participants (average age 53 compared to 61 in our study) and did not distinguish between disease and non-disease states. Others who performed species-level analysis comparing bladder genome samples with publicly available vaginal strains showed that there was an overlap of 23 species between the vagina and the bladder (Thomas-White et al., 2018). However, the majority of the samples in their study were from unrelated individuals in a less well-defined clinical population. Our observation that there is higher overlap in bacterial genera between bladder and vagina in women with UUI compared to controls and that the dominant bacteria involved in this overlap differs between these two groups indicates the possibility for more seeding of potentially pathogenic bacteria from the vagina into the bladder in women with UUI compared to controls. How this contributes to dysbiosis of the entire ecosystem is unclear.

The main limitation of our study is the small sample size that made it difficult to identify all relevant clinical and demographic factors that could influence microbiome community structure. For example, use of various vaginal products could potentially influence vaginal microbiome in different ways. Although there were no women who reported symptoms of vaginal infection in our study, we did not do objective screening for the presence or absence of infection in asymptomatic participants. We also used amplicon sequencing of the V4 region of the 16S rRNA gene to investigate the bladder and vaginal microbiomes. While this method is widely used for microbiome studies, it has known biases and limitations (Caporaso et al., 2012; Karstens et al., 2018; Knight et al., 2018). With this approach, we were limited to genus-level information, and could not robustly assess species-level differences or interactions, which may be of importance for understanding the associations to bladder health of the urogenital microbiome. One strength of our study is the use of a clinically well-characterized and demographically well-matched population of UUI and control subjects and the use of catheter-collected samples. Another, perhaps more important strength of our study is the application of network analysis methods to better understand the microbial community structures and how they differ in the urogenital tract of women with and without UUI. To our knowledge, this is the first time this analysis method has been applied to understand microbiome-wide networks in the urogenital tract of women with and without UUI. This approach provides a powerful tool to understanding the role of microbial communities as a whole in bladder health and disease and how ecosystems may be perturbed by environmental factors.

### Conclusion

Our finding highlights the importance of using network analysis techniques to understand microbial community dynamics or interactions. The study also provides further evidence of overlap in microbiomes between proximal ecosystems like the vagina and bladder and how this may affect their role in health and disease.

## Data Availability Statement

The original contributions presented in the study are publicly available. This data can be found here: https://www.ncbi.nlm.nih.gov/sra, PRJNA793927. Code for the analyses presented in this manuscriipt can be found at: https://github.com/KarstensLab/urogenital_microbiome_networks_in_uui.

## Ethics Statement

The studies involving human participants were reviewed and approved by the Oregon Health & Science University, Institutional Review Board. The patients/participants provided their written informed consent to participate in this study.

## Author Contributions

RN Conceiving and designing experiment, writing manuscript. EL Data analysis, writing manuscript, editing manuscript. WG Assisting in study design, editing manuscript. LK data Analysis, conceiving and designing experiment, assisting in writing manuscript, editing. MA (Deceased): Assisted in designing and performing experiment. SD Performing experiment. ED Analyzing Data. All authors listed have made a substantial, direct, and intellectual contribution to the work and approved it for publication.

## Funding

This project was supported by the Society of Urodynamics, Female Pelvic Medicine and Urogenital Reconstruction Foundation – OAB Urgency Incontinence Grant 2015 (RN); the National Institutes of Health (NIH) funded Oregon BIRCWH K12 award number K12HD043488 made possible through the Eunice Kennedy Shriver National Institute of Child Health and Human Development and the Office of Research on Women's Health (LK); the NIH National Institute of Diabetes and Digestive and Kidney Diseases K01 award number K01DK116706 (LK) and the NIH National Library of Medicine training award T15LM007088 (EL). The content is solely the responsibility of the authors and does not necessarily represent the official views of the any of the funders or National Institutes of Health.

## Conflict of Interest

The authors declare that the research was conducted in the absence of any commercial or financial relationships that could be construed as a potential conflict of interest.

## Publisher’s Note

All claims expressed in this article are solely those of the authors and do not necessarily represent those of their affiliated organizations, or those of the publisher, the editors and the reviewers. Any product that may be evaluated in this article, or claim that may be made by its manufacturer, is not guaranteed or endorsed by the publisher.

## Supplementary Material

Supplementary Figure 1 Principal Coordinate Analysis (PCoA) for the vaginal microbiome. **(A)** PCoA using Bray-Curtis distance; **(B)** PCoA using unweighted UniFrac distance; **(C)** PCoA using weighted UniFrac distance.

Supplementary Figure 2 Principal Coordinate Analysis (PCoA) for the urinary microbiome. **(A)** PCoA using Bray-Curtis distance; **(B)** PCoA using unweighted UniFrac distance; **(C)** PCoA using weighted UniFrac distance.
