# Supplementary figures and images for: Network-Based Differences in the Vaginal and Bladder Microbial Communities Between Women With and Without Urgency Urinary Incontinence
Source: Front Cell Infect Microbiol. 2022 Mar 24;12:759156. doi: 10.3389/fcimb.2022.759156 (PMC8988226; doi:10.3389/fcimb.2022.759156)

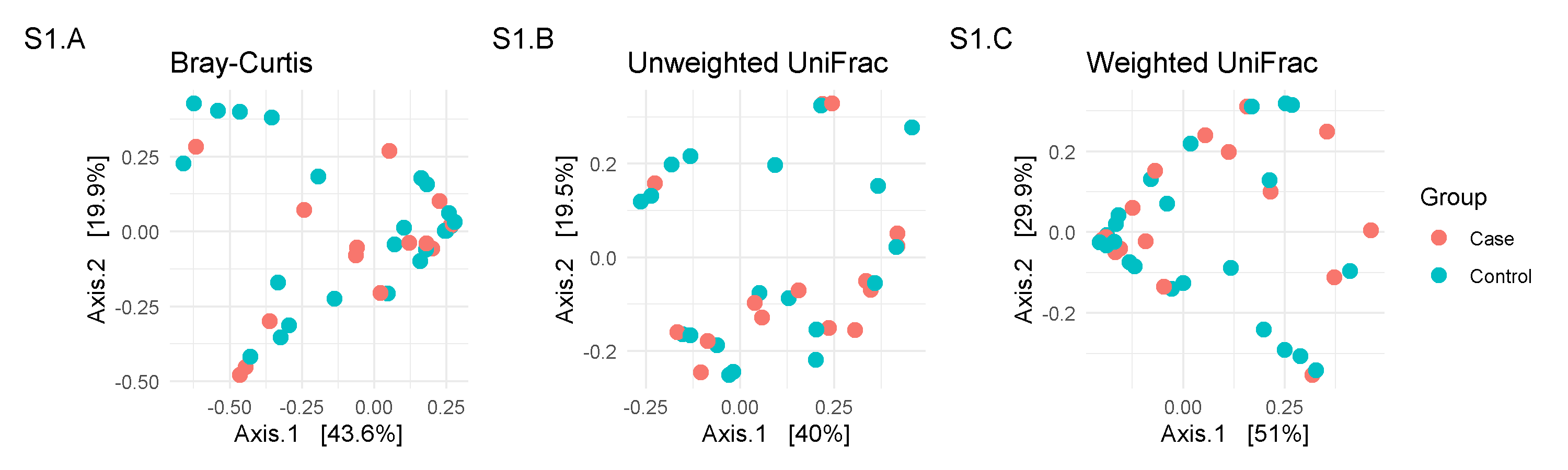

Supplement: Supplementary Figure 1 — Principal Coordinate Analysis (PCoA) for the vaginal microbiome. (A) PCoA using Bray-Curtis distance; (B) PCoA using unweighted UniFrac distance; (C) PCoA using weighted UniFrac distance. [file Image_1.tiff]

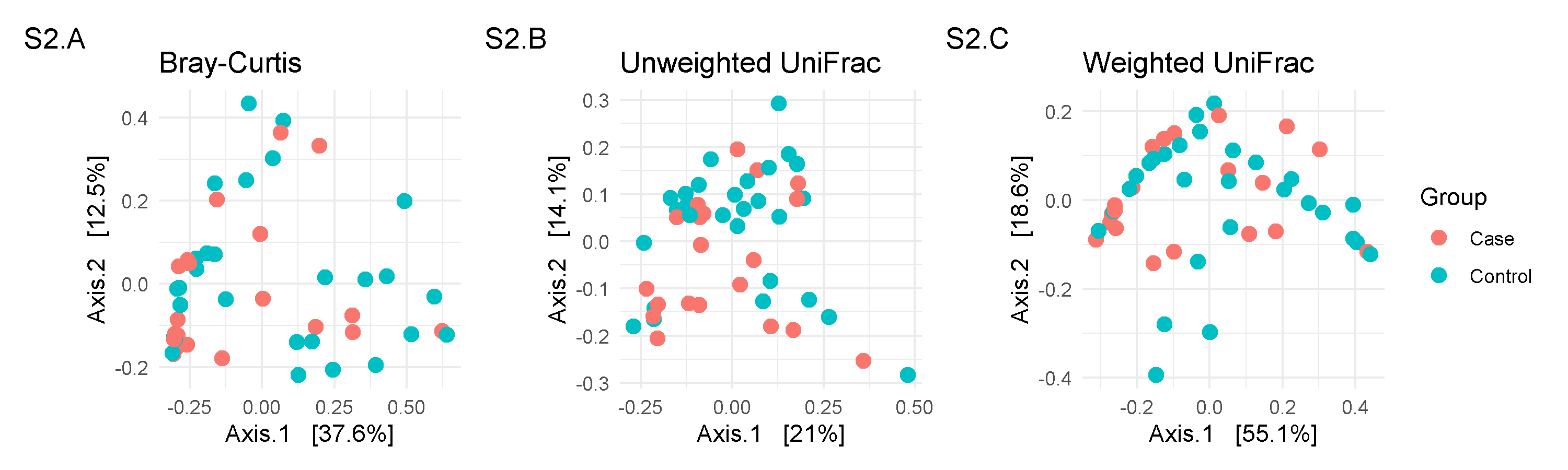

Supplement: Supplementary Figure 2 — Principal Coordinate Analysis (PCoA) for the urinary microbiome. (A) PCoA using Bray-Curtis distance; (B) PCoA using unweighted UniFrac distance; (C) PCoA using weighted UniFrac distance. [file Image_2.tiff]
